# Qualitative website analysis of information on birth after caesarean section

**DOI:** 10.1186/s12884-015-0614-0

**Published:** 2015-08-19

**Authors:** Valerie L. Peddie, Natalie Whitelaw, Grant P. Cumming, Siladitya Bhattacharya, Mairead Black

**Affiliations:** Division of Applied Health Sciences, School of Medicine and Dentistry, University of Aberdeen, Aberdeen Maternity Hospital, Cornhill Road, Aberdeen, AB25 2ZD UK; Aberdeen Maternity Hospital, Cornhill Road, Aberdeen, UK; Department of Obstetrics & Gynaecology, NHS Grampian, Dr Gray’s Hospital, Cornhill Road, Elgin, UK; Division of Applied Health Sciences, University of Aberdeen, Cornhill Road, Aberdeen, UK; Division of Applied Health Sciences, School of Medicine and Dentistry, University of Aberdeen, Cornhill Road, Aberdeen, UK

## Abstract

**Background:**

The United Kingdom (UK) caesarean section (CS) rate is largely determined by reluctance to augment trial of labour and vaginal birth. Choice between repeat CS and attempting vaginal birth after CS (VBAC) in the next pregnancy is challenging, with neither offering clear safety advantages. Women may access online information during the decision-making process. Such information is known to vary in its support for either mode of birth when assessed quantitatively. Therefore, we sought to explore qualitatively, the content and presentation of web-based health care information on birth after caesarean section (CS) in order to identify the dominant messages being conveyed.

**Methods:**

The search engine Google™ was used to conduct an internet search using terms relating to birth after CS. The ten most frequently returned websites meeting relevant purposive sampling criteria were analysed. Sampling criteria were based upon funding source, authorship and intended audience. Images and written textual content together with presence of links to additional media or external web content were analysed using descriptive and thematic analyses respectively.

**Results:**

Ten websites were analysed: five funded by Government bodies or professional membership; one via charitable donations, and four funded commercially. All sites compared the advantages and disadvantages of both repeat CS and VBAC. Commercially funded websites favoured a question and answer format alongside images, ‘pop-ups’, social media forum links and hyperlinks to third-party sites. The relationship between the parent sites and those being linked to may not be readily apparent to users, risking perception of endorsement of either VBAC or repeat CS whether intended or otherwise. Websites affiliated with Government or health services presented referenced clinical information in a factual manner with podcasts of real life experiences. Many imply greater support for VBAC than repeat CS although this was predominantly conveyed through subtle use of words rather than overt messages, with the exception of the latter being apparent in one site.

**Conclusions:**

Websites providing information on birth after CS appear to vary in nature of content according to their funding source. The most user-friendly, balanced and informative websites appear to be those funded by government agencies.

## Background

Elective repeat caesarean sections (ERCS) account for a significant portion of the overall CS rate in the UK and US [1, 2]. However, geographical variation exists in this regard with one in six pre-labour CS in England determined by previous CS, while the same indication accounted for one third of pre-labour CS in Australia [3]. The ERCS rate is affected by decisions made between couples and their health professionals in terms of whether or not to attempt a vaginal birth after CS (VBAC). As VBAC and ERCS carry differing risk profiles, and are each deemed acceptable options overall [4, 5], the availability of information on these risk profiles may play an important role in decision-making on preferred birth mode after CS. For some women, the decision to pursue VBAC may be associated with the desire to experience natural childbirth, suggesting that women may believe that successful VBAC will enhance their life experience [6, 7]. Reasons for opting for ERCS may also relate to beliefs about infant wellbeing or a desire to avoid repeating a previous birth experience [8].

The internet is known to play an important role in informing health-related decisions [9, 10]. Search engines, online symptom checkers and health checks [11] are being increasingly used by patients with the intent of contributing to positive health outcomes [12]. Women may therefore use information provided by the worldwide web (www) to help inform their decision on birth mode after CS [13]. The nature of information women receive in this manner may be influenced by characteristics of individual websites. Privately owned and commercially funded sites may have a vested interest in shaping their content as their websites may also be marketing tools. Similarly, websites may feature links to external media or webpages which imply that the index site is endorsing certain behaviours or products [12].

We observed in a previous study that the most frequently returned websites containing information on birth options after CS provide an incomplete picture of the risks involved for each approach to birthing mode when compared to the Royal College of Obstetricians and Gynaecologists (RCOG) patient information document [5]; *‘Birth after previous caesarean; Information for You’* [14]. This previous work utilised a quantitative approach to assess presence of facts which the RCOG suggest should be discussed with women planning birth after CS. However, the previous work did not allow for consideration of context in which facts were presented. The precise wording used by specific websites, the surrounding text and its allocated space, pictures, adverts or stories could each have an impact upon the readers’ perceptions - and future use - of the content. This study therefore aims to explore the content and presentation of internet-based health care information on birth after CS, focusing on the context in which information is presented, the dominant message/s being conveyed and the overall tone of relevant websites.

## Methods

Google™ (https://www.google.com), the most frequently utilised search engine worldwide, was used to perform the internet search of English language websites providing information relating to birth after previous caesarean section. Exclusion Criteria included sites relating solely to advertising or products for sale; not relating to humans (i.e. veterinary or not related to the search terms entered), and those requiring membership or payment for full access. Search terms used included those provided by a sample of women, pregnant after previous CS, attending the antenatal clinic in Aberdeen Maternity Hospital (between Dec 2013 – Jan 2014). These women were invited to participate by means of a combined invitation letter, information sheet and form containing one question regarding which search terms they would use if searching for information on birth options after CS on the internet. We received seven completed forms from 6 antenatal clinics. Ethical approval was sought, but the North of Scotland Research Ethics (NRES) Committee confirmed that this was not required. These, together with well-established keywords in this area (identified from a previous study, by Whitelaw et al*.,* [14]) were selected, and a single reviewer (NW) performed the search. The search was conducted on a personal laptop and results verified on two NHS computers. The first 10 web pages returned for each of the 10 search terms were combined giving a*n initial sample of 100 Google search results. This provided 68 unique web pages from 44 individual websites (35 if all NHS websites are grouped together).* The number of times websites recurred within this initial sample, including those returned under a slightly different uniform resource locator (URL) was recorded. Predetermined purposive sampling criteria was used to select the most frequently returned sites within this sample that met the criteria (minimum of one of the following: government-funded; commercially funded; charity-funded; aimed at the general public; UK-based, US-based and one from another country to give a final sample of 10 websites for analysis (Table 1).

**Table 1 Study sample of websites:** ᅟ

Website number	^a^Exact URL and re-occurrence (+)	URL	Funding Source
1	6 (+2)	http://www.nct.org.uk/birth/vaginal-birth-after-caesarean-vbac	Commercial
2	5 (+6)	http://www.babycentre.co.uk/a557727/vaginal-birth-after-caesarean-vbac	Commercial
3	4 (+4)	http://www.nhs.uk/conditions/caesarean-section/pages/introduction.aspx	Government
4	4 (+4)	http://www.nhs.uk/conditions/pregnancy-and-baby/pages/caesarean-section.aspx	Government
5	3 (+3)	http://en.wikipedia.org/wiki/Vaginal_birth_after_caesarean	Commercial
6	3 (+3)	http://www.netmums.com/pregnancy/labour-and-birth/about-the-birth/vaginal-birth-after-caesarean-section-vbac	Commercial
7	3 (+1)	http://www.rcog.org.uk/womens-health/clinical-guidance/birth-after-previous-caesarean-birth-green-top-45	Membership
8	3 (+1)	http://www.bbc.co.uk/news/health-17353803	Commercial
9	3 (+0)	http://sogc.org/guidelines/guidelines-for-vaginal-birth-after-previous-caesarean-birth-replaces-147-july-2004	Membership
10	3 (+0)	http://www.newcastle-hospitals.org.uk/services/maternity-unit_treatment-and-medication_choices-for-birth-after-a-caesarean-section.aspx	Government

^a^Website URL number relates to the number of times the exact webpage appeared across the various different searches and the one in brackets relates to the same website which appeared in the different searches but a slightly different URL, (different webpage of the same website)

The first page (excluding hyperlinks) for each of the 10 selected websites was examined systematically to assess the format and different types of information provided, together with the context (surrounding text, images and other media, eg. podcasts) in which it was presented. Website characteristics including textual and graphical (for example, images; pop-ups & podcasts) information were analysed. The webpages were read, and website text was entered into Microsoft Excel®. Content (thematic) analysis was applied by VP (qualitative researcher with clinical background in obstetrics), during which data were coded into substantive categories. For example one website opened with a bold and positive statement: ‘*If you have had a caesarean birth the options remain open for the birth of your next baby. Most women who choose ‘VBAC’ have a successful vaginal birth’,* which was subsequently categorised and coded as ‘positive portrayal of VBAC’. By comparison, statements depicting a more pro CS stance: *‘During a VBAC you can have an epidural for pain relief. However, you may choose not to have an epidural so you can be aware of early symptoms of uterine rupture…..’* were subsequently categorised and coded as ‘negative portrayal of VBAC’. Constant comparison analysis of the coded data was applied to identify additional patterns/themes and to explore disconfirming data. *Whilst the analysis was done by one member of the research team with qualitative research experience (VP), a second member of the team (MB), who also has qualitative research experience, checked a subset of codes/themes against relevant text*, and ideas or concepts that underpinned the theme or category discussed. This process facilitated appreciation of contextual aspects of website content and the dominant message (s) being conveyed. This included detailed exploration of the tone in which risks and benefits associated with birthing mode options were discussed. On occasion, the way in which information was presented, had the potential for raising anxiety levels beyond that which is arguably justified by the actual risk involved, and as a result, the theoretical ability to affect birthing mode intentions. An example includes where the expressed risk of uterine rupture appeared distorted when compared with absolute risk, or mentioning only very briefly the risks of VBAC, might suggest a pro repeat CS and pro-VBAC stance respectively. The initial coding categories were discussed among the authors, with modifications made to enrich meaning. Where specific websites have been cited, they have been identified by ‘W’ (website) and number (refer to Table 1).

## Results

The ten websites reviewed originated in three countries (UK, US and Canada) (Table 1). Five were funded by Government (Health Services) or professional membership; one via charitable donations, and four by commercial means. Those funded by Government or professional membership specified multidisciplinary, professional authorship; NHS sites are certified by the Information Standard for Health and Social Care - which was established by the Department of Health (DoH) - and declared collaboration with The Royal Society for Public Health Certification. The Charitable site and four of those commercially funded gave no clear implication regarding responsibility and accountability for published material, with one commercially funded site declaring collaborative, anonymous contributions from other internet users. The others did not declare defined quality assurance. The qualitative analysis identified four major categories reflecting website content: (1) ‘presentation style/marketing’, including use of images, hyperlinks and advertisements; (2) ‘information bias’, where wording implied support for one particular birthing mode; (3) ‘prominence of information’, for example, one site displayed a prominent central text box illustrating its overall message: ‘Birthing tip’ *‘most women who choose a VBAC succeed in having a vaginal birth’* and (4) ‘patient engagement’, where sites either promoted or played down collaborative decision-making and the role of women in planning the birth after CS*.*

### Presentation style/marketing

Differences in presentation style were identified between websites commercially funded and those affiliated with Government and/or health services. The latter favoured a factual presentation style, whilst commercially funded sites tended to utilise question and answer format with advertisements/images, although some portrayed elements of both.

One charitable site appeared to promote VBAC by providing hyperlinks to information predominately relating to VBAC (W1). This site advocated trial of labour and VBAC in the context of promoting women’s birth choices; the overall message presented in a prominent textbox captured this; ‘Birthing tip’ *‘most women who choose a VBAC succeed in having a vaginal birth’* (W1).

Commercially funded sites featured images (for example, mothers with new born babies and those of ‘family units’), hyperlinks to colourful advertisements, special offers (often related to baby products and local events), links to social media sites (including reader forums), local events, as well as information on local birthing units. One commercially funded site invited readers to ‘share’ on social media sites and encouraged real time discussion with others, uploading of their latest ‘prized’ images with the chance to enter competitions (W6).

Irrespective of funding source, most websites embraced social media, with links to email, *Facebook* and *Twitter* accounts (W2,W3,W4,W6,W7,W9); one displaying a ‘pop up’ box inviting readers to sign up to ‘*free pregnancy and baby emails’* (W4). Similarly, most contained easy to navigate links to position statements and citations which supported the primary text, frequently asked questions and further information (W1,W3,W4,W5,W6,W9,W10). This included ethical considerations, consultation documents (W7,W9) and web-based applications (‘Apps’) (7).

In support of VBAC, Government funded sites used podcasts to support discussion with healthcare professionals of clinically justified CS. Such sites also featured patient testimonials using podcasts and written text in support of VBAC, and personal experience of CS (W3,W4). These sites linked to peer reviewed publications, resources and guidelines. They supported VBAC with visual content (for example, images of family units and women breastfeeding), further suggesting quick recovery following vaginal birth. Overall, Government and professional membership sites provided a balanced view in the presentation and style of their homepage.

### Information bias (discourage (deter) vs encourage)

The way in which information was portrayed, and the language used in several websites appeared biased in support of or against VBAC/ERCS. Some sites adopted the ‘persuasive’ (positive) approach in advocating trial of labour, discussing VBAC as the default position (W7,W9,W10); ‘*Provided there are no contraindications, a woman with 1 previous transverse low-segment Caesarean section should be offered a trial of labour after Caesarean with appropriate discussion of maternal and perinatal risks and benefits’* (9) and *‘three out of four women will go on to have a vaginal delivery’* (W10). Where content relating to trial of labour with minimal intervention, minimal risk (W1,W3,W7,W9,W10), and successful outcome of VBAC (W1,W5,W6,W9,W10) was given greater prominence, this was perceived as ‘positive information provision’: *‘if you have a baby by caesarean section, this does not necessarily mean that any baby you have in the future will have to be delivered by caesarean section’* (W4), and ‘*attempts at vaginal birth after caesarean (VBAC) have a high success rate and have many benefits’* (W9).

On the other hand, some websites appeared to support repeat CS by emphasising the associated risks with trial of labour; *‘during a VBAC you can have an epidural for pain relief. However, you may choose not to have an epidural so you can be aware of early symptoms of uterine rupture…..’* (W2), which was subsequently perceived and termed ‘negative information provision’. One website appeared to have difficulty in providing a balanced view when citing data from ‘studies’ through apparent misleading representation of the absolute risks; *‘The UK research is the first to compile national data about the risk of womb rupture - a serious complication of pregnancy, which can cause severe blood loss in the mother and put the baby at risk’* (W8). The frequent use of medical terminology with limited information to support the ‘research’ findings quoted, further suggests that online information has the potential for misinterpretation; ‘in women who had a previous C-section, the risk of the womb rupturing during labour was seven times higher if they tried for a natural labour, compared with a planned C-section. The risk of the baby dying was three times higher’. However, as stated in a proceeding statement; ‘the overall risk was low - 2 in 10,000 of every UK pregnancies’ (W8), thus, an astute reader could make an objective assessment of the risk involved, but a casual reader may perceive risk of VBAC too great to contemplate. Similarly, a commercially funded site appeared to highlight and repeat the risks associated with VBAC; namely the 25 % risk of emergency CS and potential requirement for blood transfusion and risk of uterine infection associated with VBAC. In an apparent attempt to redress the balance, it went on to say; *‘so if your VBAC is successful*, *you’ll have a lower risk of some minor and major complications’* (W2).

One commercially funded site provided an apparently balanced view by discussing the impact of historical data on current clinical practice, whereby the United States (US) was described as coming full circle in the context of previous collaborative support of repeat CS; *‘the American College of Obstetrics and Gynaecology subsequently issued guidelines which identified VBAC as a high-risk delivery requiring the availability of an anesthesiologist, an obstetrician, and an operating room on standby’,* subsequently leading to a radical drop in *VBAC cases’* (W5)*.* However, it went on to describe the cultural transference that resulted following combined efforts, which echoed the philosophy of the Royal College of Gynaecologists (RCOG) and demonstrated; *‘widespread public and professional concern about the increasing proportion of births by caesarean section’* (W7), thus *‘enhanced access to VBAC has been recommended based on the most recent scientific data on the safety of VBAC as compared to repeat caesarean section….’* (W5)*.*

### Prominence of information

Some gave greater prominence – through physical space on their webpage - to negative outcomes (uterine rupture and infection; fetal death) in attempting VBAC; one commercially funded site in particular highlighted the potential for *‘long term problems associated with uterine prolapse and stress incontinence and perceiving VBAC as high risk for subsequent emergency CS (25 %) with need for blood transfusion’* (W2). Another commercially funded site gave prominence to the potential risk of fetal death during VBAC; *‘the chance of your baby dying in labour is increased slightly with a VBAC compared to if you were to have a caesarean delivery’*. However, the authors later engage an understanding of relative risk in this context; ‘*the rate is actually the same as what it is with a first time mum (1:10000) so is only a very slight risk’* (W6). Government funded sites attempted to provide a balanced argument, portraying risk as a cumulative incidence (a measure of risk occurring over period of time) in addition to simple language, which suggested these risks as non-emergency in nature; *‘if your midwife and doctor are concerned about the safety of you or your baby, they will suggest that you have a caesarean straight away, for instance if your cervix doesn’t dilate fully during labour and birth isn't progressing properly, or if you bleed during labour’* (W4). However, simple language was used by most to communicate the risks associated with birthing mode (W1,W3,W4,W5,W6,W9,W10). One commercially funded site gave prominence to results of several unreferenced research studies, moving seamlessly and selectively from one ‘study’ to another; *‘Australian researchers found the risk of stillbirth was lower in women who had a planned repeat C-section rather than trying for a natural labour’* and *‘the team from the National Perinatal Epidemiology Unit at Oxford University identified 159 cases of womb rupture between April 2009 and April 2010, with the vast majority of cases - 139 - in women who had already had a Caesarean’* (W8). However, contradiction was apparent in its overall less prominent message; *‘choosing a vaginal birth or a caesarean section carries different risks and benefits but overall either choice is safe with only very small risks’* (W8), which had the potential for being overshadowed by the preceding text. Government sites gave greater prominence to the positive perception of VBAC; *‘According to the American Pregnancy Association, 90 % of women who have undergone caesarean deliveries are candidates for VBAC’* (W7).

### Patient engagement

Irrespective of funding source, most websites advocated patient autonomy, choice and involvement in decision-making (W1,W3,W4,W7,W9,W10). One charitable site promoted VBAC by tapping into women’s desire for ‘minimal intervention’ (W1), and another highlighted the psychological benefits of trial of labour and VBAC; *‘vaginal delivery is the most natural way to give birth and you should not underestimate the value of this experience’* (W10). An NHS Trust-specific webpage suggested women ‘*consider their thoughts about how they want to deliver their baby’* (W10), whilst at the same time, acknowledging the appropriate clinical justification for CS where a repeat CS might be recommended (W9), referring to first or subsequent pregnancies. One site provided links to news podcasts and review of the National Institute for Clinical Excellence (NICE) guidelines, appearing to advocate a patient centred approach and maximising autonomy and choice in the context of mode of birth (W9). Government funded sites proposed that recommendation be based on the best available evidence (W3,W4,W7,W9,W10); *‘ask your health care professional if VBAC is right for you‘* and *’your doctor will review your surgery record and discuss whether a trial of VBAC is right for you'* (9), thereby defining patient engagement as a collaborative effort and in-depth discussion with healthcare professionals.

## Discussion

This study established that most websites provided correct and balanced information based upon our clinical knowledge and the content of the RCOG document *‘Birth after previous caesarean; Information for You’* [5]. In addition, all websites compared the advantages and disadvantages of both repeat CS and VBAC. Government funded websites advocated a shared decision-making approach, providing balanced and referenced information in the context of VBAC, and information largely presented VBAC as the ‘default position’ in the absence of contraindications to CS. This was consistent with our previous work which demonstrated a trend towards government-funded sites displaying more information in support of VBAC than commercial sites [14]. Most sites, but in particular, commercially funded sites, incorporated advertisements, pop-ups, and links to online forums and social media, which has the potential for bias in leading women to personal opinions of experiences.

The majority of websites included detailed accounts of potential risks of VBAC (maternal and fetal mortality), with non-government funded sites containing occasionally misleading information, which may influence women’s perception of the absolute risk/s. Internet based information (Web 1.0 technologies) provides a convenient forum/venue for the publication of information relating to health. Innovative features (Web 2.0 technologies), such as online forums, afforded patient interaction and the opportunity to respond to comments or consultations in a way that may not be possible with their health care professionals [13]. Nevertheless, in relation to healthcare and in particular, pregnancy and birthing mode, it is important to assess the nature of information contained within the websites and its quality assurance. Our findings suggest that the presentation style of websites differ in relation to funding source. Government-affiliated sites preferred a factual style, whereas commercially funded sites tended to feature integration of health related information with links to additional web content (advertisements, offers and ‘social media’ forums), which may be indicative of an attempt to influence consumer behaviour. In addition, navigational challenges – such as links to parent sites - might also distort the overall healthcare message contained within. All sites reported the risks and benefits associated with VBAC, however, presentation of information contributes to the overall message being conveyed, thus the way in which risk is portrayed or illustrated, may serve to negate the message intended. Overall, most sites attempted to present a balanced viewpoint, however presentation style, context and prominence given to specific information/graphical text implied greater support for one specific birthing mode. Whilst Weston and Anderson [13] explored generic internet use in pregnancy, to our knowledge, no other study has used qualitative methods to explore the content and context of web-based information on birth after CS.

Pregnant women use the internet to pursue social support, compare experiences and search for information [15, 16]. Forum engagement is recognised as increasing inter-connectivity amongst individuals [17], whilst reinforcing the need for women to be in control of their birthing choices. Because the majority (75 %) of Internet users who look online for health advice do not consistently check the source and date of posting of information [18], a potential drawback includes the lack or subjective nature of evaluating the quality of web-based information. Yet, over half of online users discussed their findings with health care professionals [19]. Subsequently, there exists the potential for concern, as women’s expectations of healthcare are likely to evolve in response to Internet-use [20], and the potential for disappointment if these are not met [21].

It is apparent that, the way in which information is presented - including the language used -may create information bias. The multiple means by which online information is presented may be an advantage to some women, in that information may need to be ‘seen’ in visual form (images/podcasts) to be understood, which concurs with findings from previous studies [22, 23]. Whilst healthcare professionals exert influence on women’s decisions; life experience and attitudes underpin our understanding of risk-based decision-making [8].

Some text was open to multiple interpretations. Thus, ambiguity was evident in the context of presentation of ‘statistics’; with popular media further complicating the decision-making process through sensationalist headlining and biased reporting of health related studies. There is the potential therefore, for contradiction, which may result in the intended message being lost. Chou and colleagues [17] identified with this finding, in the *‘indirect and unintended negative health impacts’* of media reporting and online forum activity. As a result, women might use online information to assess the probability and subsequent cost or benefit associated with different birthing modes. Women’s views of the personal relevance and perceived accuracy of online information create the potential for regret, for example women may select ERCS over trial of labour and later encounter a sense of failure and dissatisfaction as a result [24].

The decision-making process in pregnancy, specifically information relating to birthing mode can be complicated and overwhelming, and this is further complicated by the vast number of websites in existence. The literature also suggests that women are more likely than men to seek online diagnoses and advice [19], therefore need to manage the influence of these factors on decision-making in deciding the best course of action. Understanding the complexity of information provided requires key capabilities; cognitive, reflexive and social [24]. Therefore healthcare professionals need to consider both the clinical and social implications, which might further influence women’s decision-making in the context of birthing mode.

Whilst the websites accessed were from the UK, US and Canada, we cannot distinguish their relevance for other countries, but suggest that the content is applicable across the developed world where public health is the norm. The National Health Service (NHS) advocate patient autonomy and shared decision-making in treatment choices [23, 25, 26], and the content and context of web-based information may indeed play a vital role in this process [19]. Although practitioners acknowledge the influence of media resources on women’s attitudes towards pregnancy and birthing mode, equally consumers may be empowered through increased knowledge and information [19, 26, 27], irrespective of source or quality. Nevertheless, our recommendation is that women seeking balanced and evidenced-based information should give priority to government-funded sites.

### Strengths and limitations of the study

Adopting a qualitative approach to assessing the content of web-based information on birth after CS enables a greater understanding of both overt and subtle messages portrayed by websites likely to be accessed by women searching for information on this topic. Paying attention to both textual and graphical content ensures a broad appreciation of the impact a website may have on readers, enhancing previous analyses of quantitative nature. Due to restricting our analysis to the first webpage of a purposive sample, may mean that key content is not analysed or that other frequently accessed websites were not included in the study. These possibilities were each considered unlikely to undermine the study findings, as the purposive sampling strategy did not result in removal of any of the ten most frequently accessed sites from the final sample, while the content analysis of the first webpage was considered highly likely to encompass the content that women actually read. Finally, we cannot distinguish with confidence, the relevance of British, American and Canadian web-based information in other countries, where the information might be applicable, yet limited in access, due to tight control or censorship.

### Future research

Knowledge of the impact that web-based information on birth after CS has on women’s decision-making is limited. It is also unclear how use of such information impacts upon the clinician-patient relationship. Therefore, further studies are required to evaluate the direct effect of web-based information in this context.

## Conclusions

Our findings suggest disparity in the context of both quality and content of information on websites associated with birth after CS. Content appears to vary in nature according to their funding source. The most user-friendly, balanced and informative websites appear to be those funded by government agencies. The findings of this study might better inform health care professionals - in the context of web content and style - in an attempt to guide women to high quality resources and ensure informed use of these.
